# Supplementary material for: Parkin Inhibits RANKL-Induced Osteoclastogenesis and Ovariectomy-Induced Bone Loss
Source: Biomolecules. 2022 Oct 31;12(11):1602. doi: 10.3390/biom12111602 (PMC9687699; doi:10.3390/biom12111602)
Supplement: Supplementary file 1 [file biomolecules-12-01602-s001.zip › biomolecules-1937564-supplementary.pdf]

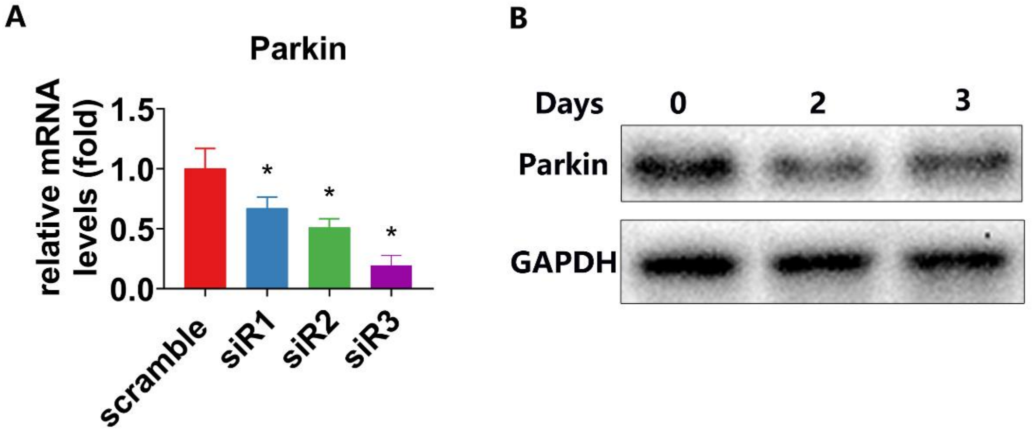

Figure S1. (**A**), mRNA levels of Parkin were analyzed by PCR at day 2 after small-interfering RNA treatment. "scramble" represents BMMs transfected with control siRNA. "siR1" represents BMMs transfected with Parkin-siRNA1. "siR2" represents BMMs transfected with Parkin-siRNA2; "siR3" represents BMMs transfected with Parkin-siRNA3; * $P < 0.05$ compared with scramble group. (**B**), Protein levels of Parkin were analyzed by WB at days 0, 2, 3 after small-interfering RNA3 treatment.

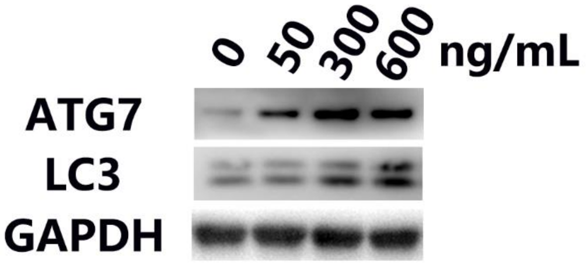

Figure S2. BMMs were pre-treated with vehicle or Parkin (50, 300, 600 ng/mL) for 6 h and thereafter exposed to RANKL (100 ng/mL) for 1 day. Then, Western blots analysis were performed.
